# Onset of Mucormycosis in Patients with COVID-19: A Systematic Review on Patients' Characteristics

**DOI:** 10.1055/s-0042-1751003

**Published:** 2022-09-01

**Authors:** Shohreh Ghasemi, Mahmood Dashti, Amir Fahimipour, Ghazaleh Daryakenari, Fatemeh Mirzaei, Fatemeh Akbari, Zohaib Khurshid

**Affiliations:** 1 Department of Oral and Maxillofacial Surgery, The Dental College of Georgia at Augusta University, Augusta, GA, USA; 2 Department of Craniofacial Reconstruction and Trauma Queen Marry, University of London, London, UK; 3 Department of Orthodontics, Georgia School of Orthodontics, Atlanta, Georgia, USA; 4 Department of Oral Surgery, Medicine and Diagnostics, School of Dentistry, Faculty of Medicine and Health, Westmead Centre for Oral Health, The University of Sydney, Sydney, Australia; 5 Department of Oral and Maxillofacial Surgery, Student Research Committee, Golestan University of Medical Sciences, Gorgan, Iran; 6 Student Research Committee, Golestan University of Medical Sciences, Gorgan, Iran; 7 Department of Dentistry, Mazandaran University of Medical Science, Mazandaran, Iran; 8 Department of Prosthodontics and Dental Implantology, College of Dentistry, King Faisal University, Al-Ahsa, Kingdom of Saudi Arabia

**Keywords:** novel coronavirus disease 2019, COVID-19-associated candidiasis, mucormycosis, corticosteroid treatment, fungal co infections

## Abstract

Mucormycosis has a significant impact on patients' standard of living and, therefore, a high clinical suspicion, prediagnosis, and rapid treatment are critical in easing patients' suffering and fast recovery. Our focus is to conduct an organized review based on various variables on the patients' characteristics having mucormycosis in severe novel coronavirus disease 2019 (COVID-19). We examined Embase, PubMed-Medline, LitCovid, Web of Science, Scopus, and the reference lists of included case reports up to September 20, 2021, using the Medical Subject Heading (MeSH) phrases and other keywords related to this topic. Subsequently, we investigated associated comorbidities, patient characteristics, position of mucormycosis, steroids use, body involvements, and outcomes. Overall, 77 studies were conducted and among these, 72 studies mentioned that the patients' age to be 48.13±14.33 (mean±standard deviation [SD]) years. Diabetes mellitus (DM) was reported in 77.9% ( *n* =60) of cases. Studies showed that central nervous system (CNS) and bone involvement were reported in 62.3 ( *n* =48) and 53.2% ( *n* =41), respectively. More fatalities were observed in patients with mucormycosis with the active form of COVID-19. Also, men infected with mucormycosis significantly affected by COVID-19. In the end, mortality was higher in males with mucormycosis. As a result, a solid investigation into the root cause of mucormycosis, especially in COVID-19, should be included in the study plan. If the patient is COVID-19-positive and immunosuppressed, this opportunistic pathogen diagnostic test should not be overlooked.

## Introduction

The World Health Organization (WHO) declared novel coronavirus disease 2019 (COVID-19) as a pandemic in March 2020, caused by severe acute respiratory syndrome coronavirus 2 (SARS-CoV-2) bottom. With more than 162 million cases and more than 3 million deaths worldwide, the pandemic continues to be a public health problem. 1

With over 24 million reported cases of COVID-19, the Indian subcontinent is second only to the United States. As the number of cases increases worldwide, the number of potential side effects of COVID-19 is becoming apparent, including an increased risk of secondary bacterial and fungal infections. 1 2

In addition, patients with COVID-19 who have decompensated lung function and who need invasive ventilation are more likely to develop a secondary infection. The rate of secondary fungal and bacterial infections in hospitals is estimated to be approximately 8%. Previous studies have shown that fungal infections are more likely to occur late in COVID-19 infection, with much higher mortality in patients with multiple fungal infections. 3 4 5 6

Mucormycosis is known to influence people with compromised immune systems, including those with diabetes, hematological malignancies, long-term corticosteroid usage, neutropenia, and solid organ transplant recipients. 5 It is an invasive infection that causes necrosis and infarction in several end-organ host tissues by allowing fungal hyphae to invade blood vessels. Even with appropriate treatment, a rhinoorbital infection caused by the fungus Mucorales has a terrible prognosis, with a death rate of up to 50%. 7

The main objective of the study was to put together the related studies regarding COVID-19 associated mucormycosis (CAM) to investigate what are the contributing factors to the outcome of mucormycosis in COVID-19 patients. We have included every case report, case series, and observational studies, so we have a stronger database of cases.

## Method

### Protocol and Registration

The report for this study was based on the Preferred Reporting Items for Systematic Reviews and Meta-analyses (PRISMA) checklist. 8 Using the Population, Intervention, Comparison, Results, Study Design (PICOS), the clinical questions were identified.

PICO entailed the following:

- Patient: CODID-19-positive patients.
- Intervention: mucormycosis fungal infection in the head and neck region.
- Comparison: patient history, drugs received, outcome, and central nervous system (CNS) involvement.
- Outcome: how much has the fungal infection spread and the outcome of the disease.

### Inclusion Criteria

Case reports, case series, and observational studies were included. Literature, excluding review studies, focusing on the spread of mucormycosis disease infection in patients infected with the SARS-CoV-2 virus was also included. There was no limit to the year of publication. It contains articles published in English and the result variables are as follows:

- Patients with SARS-CoV-2 who have mucormycosis fungal infection.
- Mucormycosis in the head and neck region.
- Case reports, case series, and observational studies.

### Exclusion Criteria

Primary or proprietary clinical studies, summaries, animal studies, *in vitro* studies, stories, conference papers, other types of nonsystematic reviews (e.g., critical reviews, summaries, and state-of-the-art reviews) and *in vitro* reviews on animal studies, systematic reviews, and meta-analyses have been excluded.

### Information Sources and Search Strategy

We searched the following electronic databases using the Medical Subject Headings (MeSH) terms and other keywords related to this topic (until September 20, 2021): PubMed-Medline, Embase, Scopus, LitCovid, Web of Science, and more. We have manually searched and identified a case report reference list that has been identified potentially related document. EndNote X20 and Thomson Reuters were used to manage references. The terms used to conduct the search are shown in Table 1 .

**Table 1:** Search terms included the following

Database	Strategy of search	Results
PubMed	(“Mucormycosis”[Mesh]) AND Covid-19	42
Embase	mucormycosis:ti,ab,kw AND ('coronavirus disease 2019':ti,ab,kw OR covid19:ti,ab,kw OR 'sars-cov-2':ti,ab,kw)	79
LitCovid	Mucormycosis	146
Web of Science	TS=(Covid19ORSars-cov-2) AND TS=Mucormycosis Indexes=SCI-EXPANDED, SSCI, A&HCI, CPCI-S, CPCI-SSH, BKCI-S, BKCI-SSH, ESCI, CCR-EXPANDED, IC Timespan=All years	29
Scopus	(TITLE-ABS-KEY (sars-cov-2) OR TITLE-ABS-KEY (covid19) OR TITLE-ABS-KEY (“Corona virus”)) AND (TITLE-ABS-KEY (mucormycosis))	59
Scopus Secondary document	(TITLE-ABS-KEY (sars-cov-2) OR TITLE-ABS-KEY (covid19) OR TITLE-ABS-KEY (“Corona virus”)) AND (TITLE-ABS-KEY (mucormycosis))	1

### Study Selection

The search for the appropriate study was conducted independently by two reviewers (F.M. and F.A.), and inclusion and exclusion criteria were used to determine the appropriate case report and case series. The two reviewers read the title and/or summary to select a potentially appropriate study. If necessary, the validity of the full-text article was evaluated. Discrepancies between reviewers have been resolved through a third party.

### Data Extraction

Based on a predefined checklist worksheet, the following data were collected from articles by two authors (F.A. and F.M.) and supervised by two other authors (S.G. and M.D.).

*Summary of case report and series features* : author's name and year of publication, location of the report, number of cases, age and gender, comorbidities, COVID-19 status (active/curative), received for COVID-19 treatment, confirmation or suspected mucormycosis, site of mucormycosis, and outcome were obtained.

## Results

**Fig. 1:**
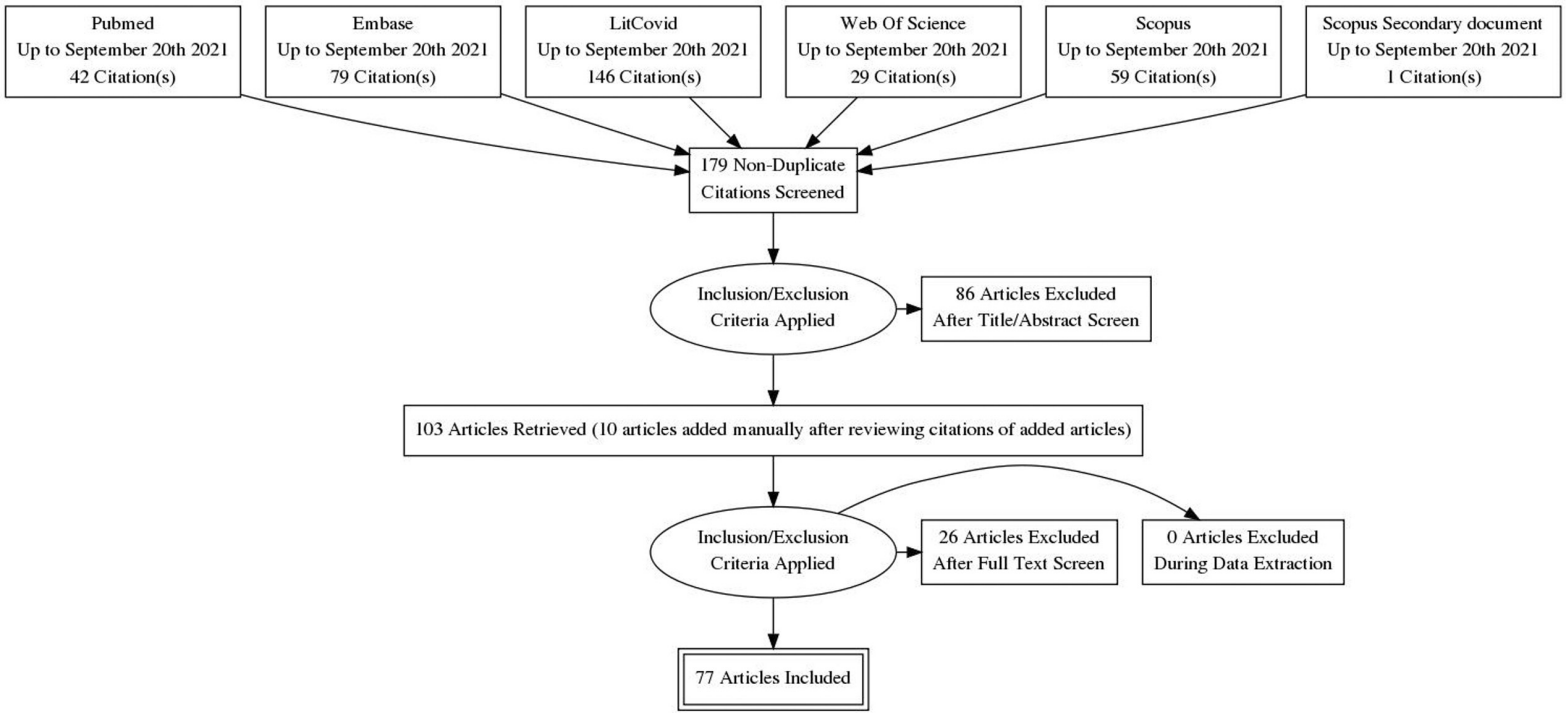
PRISMA flow diagram. PRISMA, Preferred Reporting Items for Systematic Reviews and Meta-analyses.

Based on Fig. 1 , a total of 356 studies were found from databases and 1 from other resources. After removing duplicates ( *n* =179), the remaining articles were evaluated according to their title/abstract, after which 86 articles were removed, and 93 studies remained. From the remaining 93 articles and 10 studies added later, 26 studies were excluded based on the reasons mentioned in Table 2 , resulting 77 studies included for analysis.

**Table 2:** Reasons of exclusion

Reason of exclusion	Number of excluded articles
Review article	7
Not proper location	12
Not mentioned the mucormycosis patients separately	3
Full text not related	4

Table 3 shows the 77 studies. From the 77 studies, 63.6% ( *n* =49) were from India, followed by 7 studies from Iran, 5 from the United States, 3 from Egypt, and 2 from Pakistan. Other countries such as Brazil, Colombia, Iraq, Italy, Mexico, the Netherlands, Romania, Singapore, Spain, Turkey, and the United Kingdom reported one case each.

**Table 3:** Data extraction of the 77 included studies

Study (year)	Place of report	*n*	Age (range) M/F	Comorbidities		Confirmed/Suspected COVID-19 (active/recovered)	Treatment received for COVID-19		Location of mucormycosis		Outcome
				DM	Other I.S		Steroid	Remdesivir	CNS	Bone	
Alekseyev et al (2021) 20	The United States	1	40, M	Y	N	Confirm, A	Y	N	Y	N	Recovered
Alfishawy et al (2021) 25	Egypt	21	32–72 M: 14 F: 7	5	Rhinoorbital disease: 10, rhino cerebral disease: 5 pulmonary disease: 1	Confirm, A	N	N	1	Rhinocerebral: 5	Death: 7
Arana et al (2021) 26	Spain	2	M: 62 F: 48	Y	Kidney transplantation: both	Confirm, A	Y	N	N	Musculoskeletal: 1	Recovered
Arjun et al (2021) 27	India	10	30–70 M: 8 F: 2	All	HTN: 2, hypothyroidism: 1, CAD: 3, CKD: 1, RA: 1, supplemental O2 required: 8, steroid therapy: 8, broad spectrum AB: 8, stem inhalation: 9	Confirm, A	8	N	N	N	Death:1
Ashour et al (2021) 28	Egypt	6	41–67 M: 3 F: 3	5	ESRD: 1	Confirm, A	N	N	Y	Y	Death: 2
Awal et al (2021) 29	India	3	F: 65 F: 45 M: 36	Y: 1	N	Confirm, A	N	Case 2	Y: 1	Y	N/A
Baskar et al (2021) 30	India	1	M: 28	N	N	Confirm, A	N	N	N	N	Recovered
Bayram et al (2021) 31	Turkey	11	M: 2 F: 9	N	Chronic renal failure: 3, acute renal failure: 2	Confirm, A	Y	N	Cerebral:3	N	Death: 2
Bonates et al (2021) 32	Brazil	1	M: 56	Y	N	Confirm, A	N	N	Y	N	Death
Buil et al (2021) 33	The Netherlands	4	Late 50s-late 70, M: 4	Y: 2	Chronic lymphocytic leukemia: 1, steroid therapy: 1	Confirm, A	Y	2	Y: 1	No	Death: 3
Chauhan et al (2021) 34	India	1	44, M	Y	N	Confirm, A	Y	N	N	N	Death
Dallalzadeh et al (2021) 15	The United States	2	M: 36 M: 48	2	N	Confirm, A	2	2	Y	N	Death: 1
Dave et al (2021) 35	India	58	Mean: 55±11, M: 44, F: 14	56	N	Confirm: all	37	N	Y: 19	N/A	Death: 20
Desai et al (2022) 36	India	100	30–80, M: 64 F: 36	80	HTN: 33, IHD: 9, CKD: 5	Confirm	N/A	N/A	N	Hard palate: 45	Death: 20
Desai et al (2021) 37	India	50	23–73, M: 29 F: 21	41	HTN: 17	Confirm, A	42	27	Cavernous sinus: 19	Hard palate: 15, skull base: 8	Death: 50
Eswaran et al (2021) 38	India	1	M: 31	N	N	Confirm	Y	N/A	Y	Y	Recovered
Avatef Fazeli et al (2021) 39	Iran	14	46–87 M: 6 F: 8	11	HTN: 8, CKD: 2	Confirm	N	4 cases	N	N	Death: 8
Fouad et al (2021) 40	Romania	12	16–69 M: 6 F: 6	10	CKD: 3, ALL: 2	Confirm: 6 cases	All	N	Y: 6	Y: 2	Death: 6
Garg et al (2021) 41	India	7	28–70, M: 5 F: 2	All	N	Confirm: all	Y: all	N	N	N	All recovered
Gupta et al (2021) 42	India	70	20–75, M: 47 F: 23	All	AML: 2, renal transplanted: 5	Confirm	N/A	N/A	Y	Palate: 11	Death: 4
Gupta et al (2021) 43	India	1	F: 73	Y	HTN, asthma	Confirm	N	N	N	Y	N/A
Gupta et al (2021) 44	India	59	12–78 M: 38 F: 21	41	Renal failure: 5	Confirm: all	Y	N	2	Y	Death: 4
Kumar Gupta (2021) 45	India	1	65, F	Y	N	Confirm	Y	N	N	Y	N/A
Hooli et al (2021) 46	India	10	35–70 M: 8 F: 2	8	IHD: 2	Confirm	Y	N	Y	Y	Recovered: 10
Jy Ong et al (2021) 47	Singapore	2	F: 71 M: 49	N/A	N/A	Confirm	N/A	N/A	N	Y	Recovered
Karimi-Galougahi et al (2021) 48	Iran	1	F: 6	N	N	Confirm	Y	Y	N	N	Recovered
Kaur et al (2021) 49	India	1	F: 21	Y	N	Confirm	N	N	N	N	Recovered
Krishna et al (2021) 50	The United Kingdom	1	M: 22	N	Hypothyroidism	Confirm	Y	N	Y		Death
Kumari et al (2021) 51	India	20	35–67 M: 11 F: 9	16	N/A	Confirm	Y: 16	N	Y: 4	Y: 4	Death: 6
Maini et al (2021) 52	India	1	M: 38	N	N	Confirm	Y	Y	N	N	Recovered
Mehta and Pandey (2020) 53	India	1	M: 60	Y	N	Confirm, A	Y	N	N	N	Death
Mekonnen et al (2021) 19	The United States	1	M: 60	Y	Asthma, HTN, hyperlipidemia,	Confirm, A	Y	Y	N	N	Death
Meshram et al (2021) 54	India	2	25–47 M: 2	Y: 1	HTN: 2 Transplant: 2	Confirm, R	N/A	N/A	N	N	Death: 2
Mishra et al (2021) 55	India	32	58.28 (±8.57), M: 15 F: 17	Y: 28	CAD: 2, HTN: 16	Confirm, R: 21, A: 11	Y: 30	N	N	N	Death: 4 Recovered: 28
Mitra et al (2022) 56	India	32	57±13, M: 23 F: 9	Y: All	HTN: 6, CKD: 1	Confirm: all	Y: 25	N	Pterygopalatine±Infratemporal fossa: 26	Palate: 8 Vidian canal±Pterygoid wedge: 16 Facial/eyelid soft tissues: 18	N/A
Moorthy et al (2021) 57	India	18	35–73 M: 15 F: 3	Y: 16	N/A	Confirm: all	Y: 16	N	Y: 9	Maxillary necrosis: 14	Death: 6, recovered: 11
Nair et al (2021) 58	India	13	20–51 M: 9 F: 4	N	N	Confirm	Y: 10	Y: 3	N	N	Recovered: all
Nasir et al (2021) 59	Pakistan	9	33–86 M: 6, F: 3	Y: 6	CKD: 1, chemotherapy: 2, HTN: 3, severe pulmonary HTN: 1, IHD: 3, COPD: 1, AML: 1	Confirm, A	Y: 6	N	Y: 3	N	Death: 7 (N/A)
Nehara et al (2021) 60	India	5	33–86 M: 1 F: 4	Y: all	HTN: 2	Confirm: all	Y: 3	Y: 2	Y: 2	Y: N	Death: 2 Recovered: 3
Ostovan et al (2021) 61	Iran	1	F: 61	Y	HTN	Confirm, A	N	N	Y	Y	Death
Palou et al (2021) 62	Colombia	1	M: 5	N	HTN	Confirm, R	Y	N	N	Y	Recovered
Pasero et al (2021) 63	Italy	1	M: 66	N	Arterial HTN	Confirm, A	Y	N	Y	N	Death
Patel et al (2021) 64	India	187	N/A M: 150 F: 37	Y: 113	Hematological malignancy: 2, renal transplanted: 3, traumatic inoculation: 3	Confirm: all	Y: 49	N	Rhinoorbitocerebral: 44, other: 5	N	Death: 145
Pathak et al (2021) 65	India	1	M: 65	Y	HTN	Confirm, R	N	N	N	Y	Recovered
Ramaswami et al (2021) 66	India	70	Mena: 44.5 M: 42 F: 28	Y: 49	HTN: 17 CAD: 4 CKD: 6	Confirm, A: 53 R: 17	Y: 49	N	Y: 8	N	Recovered: all
Rao et al (2021) 67	India	1	M: 66	Y	N	Confirm, A	Y	N	Patency of the intracranial vasculature	Osteomyelitis	N/A
Revannavar et al (2021) 68	India	1	Middle aged, F	Y	N	Confirm, A	N	N	Y	Y	Recovered
Roopa et al (2021) 69	India	1	F: 59	Y	N	Confirm, A	Y	Y	N	Y	Recovered
Roushdy and Hamid (2021) 70	Egypt	4	59–80 M: 3 F: 1	Y: 4	HTN: 3, IHD and cardiac, stenting: 1, operated cancer colon: 1	Confirm, R	Y: 3	N	Y: 4	Y: 2	Death: 1 Recovered: 3
Sai Krishna et al (2021) 71	India	2	34–50 M: 2	Y: 2	HTN: 1	Confirm, A: 2	N/A	N	N	Y: 2	Recovered: 2
Saidha et al (2021) 72	India	6	29–68 M: 4 F: 2	Y: 4	HTN: 1, CKD:4	Confirm: 6	Y: 1	N	N	Y: 3	Death: 1 Recovered: 5
Saldanha et al (2021) 73	India	1	23, F	Y	N	Confirm, A	N	N	N	Y	Recovered
Sarkar et al (2021) 74	India	10	23–67 M: 8 F: 2	Y: 9	CRAO: 6	Confirm: all	Y: 10	Y: 5	N	N	Death: 4 Recovered: 1
Selarka et al (2021) 75	India	1	M: 24	Y	N	Confirm, A	Y	N	N	Y	Recovered
Selarka et al (2021) 76	India	47	Mean age±SD: 55±12.8 M: 35 F: 12	Y: 36	HTN: 27, IHD: 6, COPD: 2, RA: 1, hypothyroidism: 2, sinusitis: 6	Confirm: all	Y: All	Y: 27	Y: 9	N	Recovered: 11
Sen et al (2021) 77	India	2826	Mean age: 51.9 M: 1,993 F: 833	Y: 2194	HTN: 690, renal diseases: 88, chronic sinusitis/otitis media: 18, bronchial asthma: 17, cardiovascular disorder: 16, cerebrovascular disease: 8	Confirm: all	Y: 2073	Y: 285	Laterality of central nervous system involvement, *n* : 2,669, Unilateral: 440, bilateral: 133, predominant central nervous system: 539, route of central nervous system: 430	Y: 146 (cribriform plate: 93, pterygopalatine fossa: 53)	Death: 305 Recovered: 1,913
Sen et al (2021) 78	India	6	46.2–73 M: all	Y: All	CAD: 2 HTN: 3	Confirm: All	Y: 6	N	Y: 5	Palatal eschar: 2	Recovered: All
Sethi et al (2021) 79	India	1	M: 55	Y	N	Confirm, R	Y	N	N	N	N/A
Shakir et al (2021) 80	Pakistan	1	M: 67	Y	HTN, IHD	Confirm, A	N	Y	Y	Y	Recovered
Sharma et al (2021) 12	India	23	N/A M: 15 F: 8	Y: 21	HTN: 14, renal failure: 1	Confirm, A: 4 R: 19	Y: All	N	Y: 2	N	N/A
Singh et al (2021) 81	India	1	M: 48	N	N	Confirm, R	Y	Y	Y	N	Recovered
Singh et al (2021) 82	India	14	5–75 M: 11 F: 3	Y: 8	HTN: 7, postrenal transplant: 1, Hypothyroid: 2, B-cell ALL: 2, bronchial asthma: 1, Ca breast: 1, CAD: 2, post-PTCA: 1, CLD: 1, HE: 1, febrile: 1, neutropenia: 1, disseminated TB: 1	Confirm: All	Y: 11	Y: 2	Rhinocerebral: 1 Paranasal sinus and cerebral: 1	Y: 1	Death: 9 Recovered: 4
Tabarsi et al (2021) 83	Iran	1	F: 50	Y	HTN	Confirm	Y	Y	N	N	Recovered
Veisi et al (2021) 84	Iran	2	40–54 M: 54 F: 40	Y: 1	N	Confirm, A: 2	Y: 2	Y: 2	Y: 1 Anterior cranial fossa	Y: Anterior cranial fossa	Death: 1 Recovered: 1
Venugopal and Marya (2021) 85	India	1	F: 53	Y	N	Confirm, R	N	N	Y	N	Recovered
Waizel-Haiat et al (2021) 86	The United States	1	F: 24	Y	Obesity	Confirm, A	N	N	N	N	N/A
Werthma-Ehrenreich (2021) 7	The United States	1	F: 33	Y	HTN, Asthma	Confirm, A	N	Y	Y	Y	Death
Cordero-Hernández et al (2021) 87	México	1	M: 25	Y	ALL, On chemotherapy	Confirm, A	Y	N	Y	N	Death
Mohammadi et al (2021) 88	Iran	1	M: 59	N	N	Confirm, R	Y	Y	N	Y	Death
Barman Roy et al (2021) 89	India	5	32–65 M: 3 F: 2	Y: 3	HTN: 2, Hypothyroidism: 1 IgA Nephropathy: 1	Confirm, A: all	Y: 4	N	N	Y: 2	Recovered: all
Budhiraja et al (2021) 90	India	155	53.2 (SD: 12.2) Male: 107 Female: 48	Y: 122	History of transplant: 7.7% Malignancy: 9.0%	RT-PCR+: 131 Other modes: 24	Y: 142	N	Rhinoocular cerebral: 33	N	Death: 26 Recovered: 129
Pandey and Wani (2021) 91	India	1	M: 30	Y	N	Confirm	Y	N/A	Y	Y	N/A
Farid et al (2021) 92	Iraq	1	M: 53	Y	HTN	PCR -	N/A	N/A	Y	Y	Death
Singh Rathore et al (2021) 93	India	1	M: 64	Y	CKD	Confirm, R	Methylprednisolone	N/A	Y	N	Death
Rostamihosseinkhani et al (2021) 94	Iran	1	M: 54	N	GIT stromal tumor	Confirm	Y	Y	Y	N	Death
Joshi et al (2021) 95	India	25	N/A	Y: 22	N/A	N/A	Y: All	N/A	Optic nerve: 7, cavernous sinus: 9, ophthalmic thrombosis: 12, intracranial: 7, pachymeningeal enhancement: 2, perineural extension: 1	PNS: 20, hard palate: 1	Death: 14

Abbreviations: AB, antibiotic; ALL, acute lymphoblastic leukemia; AML, acute myeloid leukemia; ARDS, Acute respiratory distress syndrome; CA, cancer; CAD, coronary artery disease; CKD, chronic kidney disease; CLD, chronic liver disease; COPD, chronic obstructive pulmonary disease; COVID-19, novel coronavirus disease 2019; CRAO, central retinal artery occlusion; DM, diabetes mellitus; ESRD, end stage renal disease; F, female; GIT, gastrointestinal tract; HE, hepatic encephalopathy; HTN, Hypertension; I.S, immunosuppressant; IHD, ischemic heart disease; M, male; N, no; N/A, not applicable; PNS, posterior nasal spine; PTCA, percutaneous coronary angiography; RA, rheumatoid arthritis; TB, tuberculosis; Y, yes.

In 76 studies, gender was mentioned adequately. Of these, 40.3% ( *n* =31) studies reported males and 11.7% ( *n* =9) of studies reported females, and 46.8% ( *n* =36) studies reported both male and female in their studies based on their study design which were case series or observational.

In 93.5% ( *n* =72) of the studies, age was mentioned to be 48.13±14.33 (mean±SD). From the 92.9% ( *n* =71) of the studies that mentioned the presence or absence of diabetes mellitus, it was reported in 77.9% ( *n* =60) of them.

As shown in Table 4 , treatment of COVID-19 was performed by steroid therapy, remdesivir, and tocolizumab in 68.8, 28.6, and 16.9%, respectively. In 83.1% of the studies, mucormycosis-infected patients had the COVID-19 active form while 11.7% of the studies reported recovered patients. CNS and bone involvement was reported in 62.3% ( *n* =48) and 53.2% ( *n* =41) of the studies, respectively.

**Table 4:** Frequency and percentage of cases based on treatment and body involvement

Treatment							Body involvement			
	Steroid therapy		Remdesivir		Tocolizumab		CNS		Bone	
	F	P	F	P	F	P	F	P	F	P
Yes	53	68.8	13	16.9	22	28.6	48	62.3	41	53.2
No	16	20.8	57	74.0	46	59.7	29	37.7	34	44.2
N/A	6	7.8	7	9.1	9	11.7	–	–	2	2.6
Missing	2	2.6	–	–	–	–	–	–	–	–
Total	77	100.0	77	100.0	77	100.0	77	100.0	77	100.0

Abbreviations: CNS, central nervous system; F, frequency; N/A, not available; P, percentage.

The status of patients is shown in Table 5 . Death was adequately reported in 40% ( *n* =31) of the studies, and 33.8% ( *n* =26) of the studies reported recovered patients. Also, 13% ( *n* =10) of the studies had both recovered and dead patients, since their studies were case series or observational. In 13% ( *n* =10) of the studies, no clear status was reported.

**Table 5:** Status of patients in studies

	Status	Frequency	Percentage
Valid	Recovered	26	33.8
	Death	31	40.3
	Not available	10	13.0
	Recovered and death	10	13.0
	Total	77	100.0

The Fisher's exact test did not reveal any substantial relationship between COVID-19 and diabetes, COVID-19 and its treatments, and COVID-19 and CNS or bone involvements ( *p* ≥ 0.05).

According to Table 6 , more fatalities were observed in patients with mucormycosis with the active form of COVID-19. Also, men infected with mucormycosis significantly affected by COVID-19. Finally, as shown in Table 7 , the death was more prevalent in males with mucormycosis.

**Table 6:** Relationship of status and gender with active (A) or recovered (R) form of COVID-19

			Status					Gender			
			Recovered	Death	N/A	Recovered and death	Total	Male	Female	Male and female	Total
Confirm	A	Count	21	28	7	8	64	24	8	31	63
		% within confirm	32.8	43.8	10.9	12.5	100.0	38.1	12.7	49.2	100.0
		% within status	80.8	90.3	70.0	80.0	83.1	77.4	88.9	86.1	82.9
	R	Count	4	3	1	1	9	7	1	1	9
		% within confirm	44.4	33.3	11.1	11.1	100.0	77.8	11.1	11.1	100.0
		% within status	15.4	9.7	10.0	10.0	11.7	22.6	11.1	2.8	11.8
	A and R	Count	1	0	2	1	4	0	0	4	4
		% within confirm	25.0	0.0	50.0	25.0	100.0	0.0	0.0	100.0	100.0
		% within status	3.8	0.0	20.0	10.0	5.2	0.0	0.0	11.1	5.3
Total		Count	26	31	10	10	77	31	9	36	76
		% within confirm	33.8	40.3	13.0	13.0	100.0	40.8	11.8	47.4	100.0
		% within status	100.0	100.0	100.0	100.0	100.0	100.0	100.0	100.0	100.0

Abbreviations: COVID-19, novel coronavirus disease 2019; N/A, not available.

**Table 7:** Relationship between gender and status of patients

			Gender			Total
			Male	Female	Male and female	
Status	Recovered	Count	14	4	8	26
		% within status	53.8	15.4	30.8	100.0
		% within gender	45.2	44.4	22.2	34.2
	Death	Count	14	2	14	30
		% within status	46.7	6.7	46.7	100.0
		% within gender	45.2	22.2	38.9	39.5
	Not available	Count	3	3	4	10
		% within status	30.0	30.0	40.0	100.0
		% within gender	9.7	33.3	11.1	13.2
	Recovered and death	Count	0	0	10	10
		% within status	0.0	0.0	100.0	100.0
		% within gender	0.0	0.0	27.8	13.2
Total		Count	31	9	36	76
		% within status	40.8	11.8	47.4	100.0
		% within gender	100.0	100.0	100.0	100.0

A total of 101 cases of zygomycosis were found in patients with confirmed COVID-19 (reverse transcription polymerase chain reaction [RT-PCR] diagnosis; including 6/101 in which 95/101 were confirmed and suspected). Eighty-two (81.2%) cases of mucormycosis in COVID-19 patients were reported from India, followed by 9 (8.9%) from the United States, and 2 (3.1%) from Pakistan. So far, only 19 cases (18.8%) have been recorded from other parts of the world. 9 10 11 Studies have shown that mucormycosis is more common in men (78.9%), regardless of COVID-19 activity (59.4%) or cure (40.6%). Patients who recovered from COVID-19 were discharged or hospitalized 2 weeks after diagnosis. However, there were some overlaps in the case.

## Discussion

Mucormycosis is a fungal infection that affects the brain, sinuses, lungs, and mouth, gastrointestinal tract, skin, and other organs. When mucormycosis affects the sinuses, it obstructs the nasal passages, resulting in dark or bloody secretions. Symptoms are unilateral facial pain or painful numbness localized to the cheekbones, skin thrombosis or necrotic skin lesions, eye thrombosis or necrotic skin lesions, skin thrombosis or necrotic skin lesions, worsening respiratory symptoms, and lung and chest pain. On the other hand, COVID-19 mainly affects COVID-19 patients in the eyes, mouth, and brain. 12 13

Since the outbreak of COVID-19 began, interest in secondary fungal infections has revived, and several case reports and short case studies have been published. In the Indian subcontinent, the number of cases of mucormycosis in COVID-19-infected persons is increasing rapidly. At the time of this writing, mucormycosis has been reported in a significant number of cases and is a public health problem in the rate of epidemics. Patients with COVID-19 should be aware of mucor disease. The current pandemic continues to be a major public health problem worldwide, and the combination of the two can cause significant morbidity and mortality. 14

To address this issue, the health system is rigorously strengthened by careful use of steroids, careful monitoring of the blood glucose status of COVID-19 patients, adherence to health mask-wearing rules, and regular monitoring of COVID-19 patients. For early detection of mucosal fungal diseases, increased antifungal supply and coordination between departments for effective management are required. 9

As the global COVID-19 pandemic approaches its second year, countries around the world are rushing to vaccinate their citizens in the face of new tensions. Physical separation, wearing masks, and other public health measures have reduced public acceptance. During the catastrophic second wave of the virus, daily COVID-19 infections steadily declined in India, affecting approximately 3,000,000 cases nationwide. The country had 28,252 recorded cases of zygomycosis from 28 states as of June 7, 2021. There are 24,370 patients with a history of COVID-19 and 17,601 patients with a history of diabetes. The country with the highest number of cases of mucormycosis was India with 6,329 cases. 10

Despite many cases of high infections in large cities that were supposed to provide some protection, the virus is spreading faster in India than ever before. The rate at which the virus spreads throughout India is confusing to scientists. Since the beginning of March 2021, the number of daily cases has increased. Outbreaks are currently occurring worldwide, including Brazil, France, Germany, and the United States, with an infection rate of approximately 70,000 daily. Hospitals are rushing for beds and oxygen in the aftermath of a dangerous second wave of infection. India has recently been blamed for more than half of all cases of COVID-19 and one-quarter of all deaths worldwide (WHO, 2021). 11

The number of COVID-19 cases is the highest since the onset of the pandemic, with over 175 million new cases reported weekly (WHO, 2021). WHO is currently monitoring four different types of concerns around the world. Varieties B.1.1.7 were found in the United Kingdom, mutant B.1.351 was found in South Africa, and mutant P.1 was found in Japan (WHO, 2021). According to genome surveillance data, the B.1.1.7 virus mutation from the United Kingdom is prevalent in the Indian state. In Maharashtra, a new potentially dangerous variety known as B.1.617 has taken root. It was first discovered in India at the end of last year. B.1.617 is of great interest because it shows two mutations associated with improved transmissibility and antigenic escape. It is currently found in 20 other countries. 15 16

The number of incidences of mucormycosis among COVID-19 patients is frightening, according to physicians. The majority of these patients had diabetes and were receiving steroid treatment for SARS-CoV-2 infection which could have made them more susceptible to fungal infection. Mucormycosis is more common in people who have a weakened immune system or who have had a bone marrow transplant and have less neutrophils. Patients with COVID-19 are given high doses of steroids which weakens their immune systems and makes them vulnerable to mucormycosis. In addition, steroids can raise blood sugar levels which can be difficult for people with uncontrolled diabetes. Moreover, the acidic environment created by this condition favors the growth of fungi (Mucorales). Inhalation of filamentous fungi weakens the patient's immune defense pathway. 17

Mucormycosis has also been linked to several underlying disorders that make a person more susceptible to infection. Mucormycosis cases in COVID-19 patients are on the rise in hospitals across the country, and the condition has been declared an epidemic. As a result, the present research focuses on the mucormycosis history, its related diseases, its progress in healthy people, immune-compromised people, COVID-19-positive people, numerous risk factors, and its impact on numerous organs, as well as the problems of overcoming this infection. With the COVID-19 epidemic putting a strain on health care infrastructure, this study will provide a general database for the right treatment results and the control of this fungal infection. 18

While some aspects of this thought-provoking disease emerged during this pandemic, the most important preventable factor in addressing the CAM “disease triangle” was COVID-19 with registered glycemic monitoring. It seems to be a wise and controlled use of steroids. Future research will continue to shed light on some of the nuances of the disease, but it is important to understand the dangers posed by this deadly infection and the correct and timely course. 19 20

Mucormycosis was found to be more common in immunocompromised patients (four of five), with nasal cerebrum and nasal orbital mucormycosis being the most common sites. These results are consistent with the literature results. Mucormycosis is a hematological malignancy that occurs in 90% of immunocompromised patients and is primarily associated with diabetic ketosis or neutropenia. The most common are cancers of the nose, orbit, brain, lungs, and skin. 1 2

Patients who were elderly, malnourished or had hematological malignancies had a higher rate of fatal mucormycosis. Hematological malignancy, allogenic hematopoietic stem-cell transplantation (HSCT), diabetes, and human immunodeficiency viruses (HIV) infection were all key prognostic variables in previous studies. 2 4 12 18 19 In one study, 20 age was identified as a predictive factor. Malnutrition has been established as a risk factor for mucormycosis 20 but not as a prognostic factor. The existence of two or more predisposing factors, as was shown, has a clear unfavorable impact on prognosis. 2

Mucormycosis has a severe prognosis, with mortality in the range of 17 to 51%. 14 In patients with active malignant hematological disorders, delayed diagnosis of 5 days or more and monocytopenia increase mortality. Prognosis is improved by surgical treatment in combination with antifungal agents. 2 14 Mucorales of the problematic genus or species appear to have little effect on results. 9 14 Mortality in previous studies was 65%. 4

Another factor that can contribute to the development of mucormycosis in COVID-19 patients in India is diabetes. Since India is the world's diabetic capital, indiscriminate use of steroids in diabetics may increase the number of cases of mucormycosis. Ninety-four percent of patients with COVID-19-related zygomycosis had a history of diabetic mismanagement. 12 Prolonged stay in the intensive care unit (ICU) and comorbidities, such as malignant neoplasms and posttransplant status, are also risk factors. 14 21 22

Stuffy nose, pain, and redness around the eyes and nose, headache, fever, vomiting, shortness of breath, coughing, and changes in mental status are all signs of COVID-19-related mucor disease, 14 also there are some oral manifestations, such as cutaneous symptoms and oral lesions, that may be indicator of COVID-19. 23 These sign and symptoms may vary since there might be a new unknown COVID-19 variants that the clinician is unaware of them. 24 Mucormycosis can damage the lungs but the most common sites of infection are the nose and sinuses. It can then get into your eyes and cause blindness, or it can go to your brain and cause headaches and seizures. 14 As a result, the most common type is nasal orbital mucormycosis, followed by pulmonic mucormycosis.

Concomitant medical issues, such as diabetes, critical respiratory distress syndrome, and the use of broad-spectrum antibiotics and corticosteroids are further risk factors. We see that the male-to-female ratio is higher, up to 70%, while the female-to-male ratio is lower, up to 30%. In comparison to female patients, male patients have a weak immune system. 6 14 20

Steroid treatment, remdesivir, and tocolizumab were used to treat COVID-19 in 68.8, 28.6, and 16.9% of cases, respectively. Individuals with mucormycosis had the COVID-19 active form in 83.1% of studies, while 11.7% of studies reported recovered patients. Involvement of the CNS and bones was found in 62.3% ( *n* =48) and 53.2% ( *n* =41) of the investigations, respectively. In 40% of the research, death was appropriately reported, and 10 studies featured both recovered and deceased patients because they reported more than one case.

Mortality rates for people with COVID-19 and subsequent fungal infections were found to be significantly higher (53%) than those without (31%). Despite early diagnosis and intensive surgical and medical treatment, the prognosis for recovery from mucormycosis is often poor. Our research found a similar trend.

In terms of diagnosis, the median time from COVID-19 diagnosis to the onset of mucormycosis symptoms ranged from 15.6 to 9.6 days. A 6-day delay in treatment also increases the 30-day mortality rate from 35 to 66%. 14 As a result, early diagnosis and treatment can help prevent disease progression and even death. India is one of the largest pharmaceutical centers globally but due to the rise in black fungal infections, amphotericin B, the latest treatment for mucormycosis, is deficient. 9

## Conclusion

Invasive ventilation, inadequate glycemic control, widespread reckless use of corticosteroids and broad-spectrum antibiotics, and invasive ventilation are all risk factors for mucormycosis in COVID-19 patients. Interdisciplinary approaches should include rapid diagnosis, antifungal treatment, and surgical examination and treatment that may help recover from the underlying disorder. Further investigation of zygomycosis in patients infected with COVID-19 and cured is needed. As a result, a solid investigation into the root cause of zygomycosis, especially in COVID-19, should be included in the agenda of the study. If the patient is COVID-19 positive and immunosuppressed, this opportunistic pathogen diagnostic test should not be overlooked.
